# Estrogenicity of Styrene Oligomers: Response to Ohno et al.

**DOI:** 10.1289/ehp.110-a385

**Published:** 2002-07-07

**Authors:** Ken-ichi Ohyama, Fumiko Nagai

**Affiliations:** Tokyo Metropolitan Research Laboratory of Public Health, Tokyo, Japan, E-mail: ohyama@tokyo-eiken.go.jp


### Estrogenicity of Styrene Oligomers and Assessment of Estrogen Receptor Binding Assays

Polystyrene is frequently used in resins, and the styrene dimers and trimers eluted from polystyrene have been reported to have estrogenic activity (*1*). We have performed a number of *in vitro* and *in vivo* tests [i.e., estrogen receptor (ER) and androgen receptor binding assays, thyroid hormone receptor binding assays, human breast cancer cell line MCF-7 proliferation assays (E-SCREEN), uterotrophic assays in immature and ovariectomized rats, Hershberger assays, and prolactin release assays and steroidogenesis] and found no effects of styrene dimers or trimers on sex hormones in any of these assays (*2–7*). These results are supported by Fail et al. (*8*), who reported that mixtures of styrene oligomers did not show any estrogenic activity in the immature rat uterotrophic assay and the reporter gene assay. In addition, the Japan Environment Agency referred to their studies (*9*) and removed the styrene dimers and trimers from their list of endocrine disruptors (*9*). However, Ohyama et al. (*10*) reported that high concentrations of certain styrene dimers and trimers showed estrogenic effects in an ER binding assay and in the E-SCREEN assay. Recently, several assay systems have been used to assess endocrine-disrupting effects, but a few of these assay systems can cause false-positive reactions when test compounds are at high concentrations (*11*).

To assess the accuracy of the ER binding assay system and the results of Ohyama et al. (*10*), and to ascertain the safety of styrene dimers and trimers, we used a solubility test and three ER binding assays (*12*) (Table 1). The ER binding assay, which detects the direct reactivity of ligand to a receptor, is the most standardized and simple test system for the detection of specific mechanisms of estrogenic activity.

Using the radiosotope method (Method RI) as described previously (*13,14*), we observed that styrene dimers and trimers did not show statistically significant inhibitory action against the binding of [$^3$H]-17β-estradiol ($E_2$) to ER.

We used Method A to detect the binding affinities of test samples to human ERα (hERα). Using a fluorescence polarization Screen-for-Competitor Kit ERα (Takara, Kyoto, Japan) as described by Bolger et al. (*15*), we measured the difference of polarization between fluorescence-labeled $E_2$ (ES1) bound to ER and ES1 only. Styrene dimers and trimers did not show statistically significant inhibitory action against the binding of ESI to ER in this assay.

We also used Method B, the method used by Ohyama et al. (*10*), to detect the binding affinities of test samples to the human recombinant ERα coated on the microplate by competition with fluorescence-labeled $E_2$; this was performed using the Estrogen Receptor (α) Competitor Screening Kit (Wako PC, Osaka, Japan). Styrene dimers and trimers showed weak inhibitory effect on the binding of fluorescein $E_2$ to hERα at 5 μmol/L, and their binding abilities were < 30% in this assay.

To evaluate the ER binding assays themselves, we included vitamin $D_3$, naphthalene, 5α-dihydrotestosterone, and testosterone in each of the three ER binding assays; none of these compounds bound ER *in vitro* (*13,16,17*). A cross-reaction between estrogen and androgens cannot occur *in vivo* unless the androgens are metabolized. In Method RI and Method A, these nonestrogenic compounds did not show any ability to bind to the ER. However, in Method B, these compounds showed binding affinity for the recombinant hERα coated on the microplate at such high concentrations that they did not dissolve, although the binding affinity of $E_2$ was similar in each assay. These results suggest that Method B tends to detect false-positive effects and that it is less accurate at high concentrations because of a decline of specificity to estrogen at high concentrations at which compounds do not dissolve. The manufacturer's instructions for the Estrogen Receptor (α) Competitor Screening Kit used for Method B say to "make sure there is no precipitation in the solution." Styrene dimers and trimers are so hydrophobic that their solubility is very low in the buffer solutions used in each assay. On the basis of these results, styrene dimers and trimers have no affinity for ER in Methods RI and A. Nevertheless, styrene dimers and trimers exhibited some affinity for the recombinant hERα in the Method B study, similar to that described by Ohyama et al. (*10*), but at high concentrations such that the compounds were not completely dissolved. This result is not because of the difference of sensitivity between rat ER and human ER, as shown in Method A with the use of hERα, but is caused by a decrease in specificity to estrogen because of the precipitation of test compounds.

Ohyama et al. (*10*) reported that high concentrations of styrene dimers and trimers showed proliferative activity in the E-SCREEN assay. Cell proliferation can be induced by other growth factors, although proliferation of MCF-7 cell is basically $E_2$ dependent (*18–20*), and the response to $E_2$ in MCF-7 cells varies because of the various mutation of ER (*21*). Therefore, a false-positive response might only be shown in tests using proliferation as a target. The luciferase reporter gene assay, which indicates direct gene expression reactivity through the receptor, has been considered to be a more suitable assay for evaluating estrogenicity at the cellular level because of specificity to $E_2$ response (*22,23*). Styrene dimers and trimers did not show any estrogenic effect in the E-SCREEN assay and the reporter gene assay in our previous study (*6*). In addition,

**Table 1.** Solubility and binding affinity for ER of tested compounds.

| Compounds | Solubility[a] (μmol/L) | Binding affinity for ER ($ED_{30}$) (μmol/L) Method RI | Method A | Method B |
|---|---|---|---|---|
| Estrogenic compounds | | | | |
| 17β-Estradiol | > 10 | 0.0012*** | 0.005*** | 0.001*** |
| Bisphenol A | > 10 | 5.0*** | 1.7*** | 2.0** |
| Styrene dimers | | | | |
| 2,4-Diphenyl-1-butene | 1.3 | NC | NC | > 10.0 |
| *cis*-1,2-Diphenylcyclobutane | 9.4 | NC | NC | 10.0** |
| *trans*-1,2-Diphenylcyclobutane | 4.0 | NC | NC | > 10.0 |
| Styrene trimers | | | | |
| 2,4,6-Triphenyl-1-hexene | < 0.16 | NC | NC | > 10.0 |
| 1e-Phenyl-4e-(1-phenylethyl) tetralin | < 0.16 | NC | NC | > 10.0 |
| 1a-Phenyl-4e-(1-phenylethyl) tetralin | < 0.16 | NC | NC | > 10.0 |
| 1a-Phenyl-4a-(1-phenylethyl) tetralin | 0.17 | NC | NC | > 10.0 |
| 1e-Phenyl-4a-(1-phenylethyl) tetralin | 0.16 | NC | NC | 5.2** |
| 1e-Phenyl-4a-(2-phenylethyl) tetralin | < 0.16 | NC | NC | > 10.0 |
| 1a-Phenyl-4a-(2-phenylethyl) tetralin | < 0.16 | NC | NC | > 10.0 |
| Androgens | | | | |
| Testosterone | < 10 | NC | NC | 105.0*** |
| 5α-Dihydrotestosterone | < 10 | NC | NC | 45.0*** |
| Nonestrogenic compounds | | | | |
| Vitamin $D_3$ | 0.19 | NC | NC | 100.0*** |
| Naphthalene | 100 | NC | NC | 1010.0*** |

Abbreviations: $ED_{30}$, concentration equivalent to 30% activity of 100 nmol/L $E_2$; NC, no competition for binding of labeled $E_2$. Each value represents the mean of triplicate assays. .
[a]Concentration at which test compounds are saturated. ** $p < 0.01$, *** $p < 0.001$ (vs. control, Dunnett test).

at high concentrations at which test compounds were precipitated, cells indicated an abnormal response in the luciferase activity of control plasmids and in morphology (data not shown). To construct a stable assay system, we used HeLa cells transfected with an hERα expression plasmid derived from normal human liver ERα. In this assay system, styrene dimers and trimers did not show any increase in $E_2$-dependent luciferase transcription activity. These results agreed with the result of the ER binding assay. We presume that styrene dimers and trimers had no binding affinity to ER and they did not affect $E_2$-dependent transcription.

As a result, in our comparison of three ER binding assays using estrogenic and nonestrogenic compounds, it appeared that Method RI and Method A were useful for evaluating binding affinity for the ER, but Method B, similar to the method of Ohyama et al. (*10*), tended to indicate false-positives in high concentrations in which test chemicals were insoluble; this reduced the specificity of ER to $E_2$. Based on our present results and previous reports (*2–7*), we found no endocrine-disrupting activities in styrene dimers and trimers eluted from polystyrene-containing instant noodle containers.

**Katsutoshi Ohno**
**Yukimasa Azuma**
**Katsuhiro Date**
**Shigeru Nakano**
**Toru Kobayashi**
**Yasuhiro Nagao**
**Toshihiro Yamada**
Central Research Institute
Nissin Food Products Co., Ltd.
Shiga, Japan
E-mail: k-ono@mb1.nissinfoods.co.jp

### References and Notes

1. Colborn T, Dumanoski D, Myers JP. Our Stolen Future. New York:Dutton, 1996.
2. Yamada T. Synthesis, analysis and biological evaluation of styrene oligomers. Yuki Goseikagaku Kyokaishi 57:58–64 (1999).
3. Nobuhara Y, Hirano S, Azuma Y, Date K, Ohno K, Tanaka K, Matsushiro S, Sakurai T, Shiozawa S, Chiba M, et al. Biological evaluation of styrene oligomers for endocrine-disrupting effects. J Food Hyg Soc Japan 40:36–45 (1999).
4. Yamada T, Hirano S, Kobayashi K, Sakurai T, Takagi K, Tanaka M, Nagao Y, Azuma Y, Date K, Ohno K, et al. Identification, determination and biological evaluation of novel styrene trimer in polystyrene container. Bunseki Kagaku 49:493–501 (2000).
5. Azuma Y, Nobuhara Y, Date K, Ohno K, Tanaka K, Hirano S, Kobayashi K, Sakurai T, Chiba M, Yamada T. Biological evaluation of styrene oligomers for endocrine-disrupting effects (II). J Food Hyg Soc Japan 41:109–115 (2000).
6. Ohno K, Azuma Y, Nakano T, Kobayashi S, Hirano T, Nobuhara Y, Yamada T. Assessment of styrene oligomers eluted from polystyrene-made food container for estrogenic effects in vitro assays. Food Chem Toxicol 39:1233–1241 (2001).
7. Date K, Ohno K, Azuma Y, Hirano S, Kobayashi K, Sakurai T, Nobuhara Y, Yamada T. Endocrine-disrupting effects of styrene oligomers that migrated from polystyrene containers into food. Food Chem Toxicol 40:129–139 (2001).
8. Fail PA, Hines JW, Zacharewski T, Wu ZF, Borodinsky L. Assessment of polystyrene extract for estrogenic activity in the rat uterotrophic model and an in vitro recombinant receptor reporter gene assay. Drug Chem Toxicol 21(suppl 1):101–121 (1998).
9. JEA. Strategic Programs on Environmental Endocrine Disruptors '98. Available: http://www.env.go.jp/en/pol/speed98/sp98.pdf [cited 30 April 2002].
10. Ohyama K, Nagai F, Tsuchiya Y. Certain styrene oligomers have proliferative activity on MCF-7 human breast tumor cells and binding affinity for human estrogen receptor α. Environ Health Perspect 109:699–703 (2001).
11. Nakano S, Nagao Y, Kobayashi T, Tanaka M, Hirano S, Nobuhara Y, Yamada T. Problems with methods used to screen estrogenic chemicals by yeast two-hybrid assays. J Environ Health 48:83–88 (2002).
12. The Japanese Pharmacopoeia. 13th ed. Tokyo:Society of Japanese Pharmacopoeia, 1997.
13. Blair RM, Fang B, Braham WS, Hass BS, Dial SL, Moland C-L, Tong W, Shi L, Perkins R, Sheehan DM. The estrogen receptor binding affinities of 188 natural and xenochemicals: Structural diversity of ligands. Toxicol Sci 54:138–153 (2000).
14. Laws SC, Carey SA, Kelce WR, Cooper RL, Gray LE. Vinclozolin does not alter progesterone receptor (PR) function in vivo despite inhibition of PR binding by its metabolites in vitro. Toxicology 110:1–11 (1996).
15. Bolger R, Weise TE, Evin K, Nestich S, Checovich W. Rapid screening of environmental chemicals for estrogen receptor binding capacity. Environ Health Perspect 106:551–557 (1998).
16. Swami S, Krishnan AV, Feldman D. 1α, 25-dihydroxyvitamin $D_3$ down-regulates estrogen receptor abundance and suppresses estrogen actions in MCF-7 human breast cancer cells. Clin Cancer Res 6:3371–3379 (2000).
17. Nishihara T, Nishikawa J, Kanayama T, Dakeyama F, Saito K, Imagawa M, Takatori S, Kitagawa Y, Hori S, Utsumi H. Estrogenic activity of 517 chemicals by yeast two-hybrid assay. J Health Sci 46:282–298 (2000).
18. Karey KP, Sirbasku DA. Differential responsiveness of human breast cancer cell lines MCF-7 and T47D to growth factors and 17β-estradiol. Cancer Res 48:4083–4092 (1988).
19. Soto AM, Sonnenschein C. The role of estrogens on the proliferation of human breast tumor cells (MCF-7). J Steroid Biochem 23:87–94 (1985).
20. Soto AM, Sonnenschein C, Chung KL, Fernandez MF, Olea N, Serrano FO. The E-SCREEN assay as a tool to identify estrogens: an update on estrogenic environmental pollutants. Environ Health Perspect 103(suppl 7):113–122 (1995).
21. Pink JJ, Fritsch M, Bilimoria MM, Assikis VJ, Jordan VC. Cloning and characterization of a 77-kDa oestrogen receptor isolated from a human breast cancer cell line. Br J Cancer 75:17–27 (1997).
22. Pons M, Gagne D, Nicolas JC, Mehtali M. A new cellular model of response to estrogens: a bioluminescent test to characterize (anti)estrogen molecules. Biotechniques 9:450–459 (1990).
23. Saito K, Tomigahara Y, Ohe N, Isobe N, Nakatsuka I, Kaneko H. Lack of significant estrogenic or antiestrogenic activity of pyrethroid insecticides in three in vitro assays based on classic estrogen receptor α-mediated mechanisms. Toxicol Sci 57:54–60 (2000).

### Estrogenicity of Styrene Oligomers: Response to Ohno et al.

The main point of the letter by Ohno et al. is that styrene oligomers have no estrogenic activity, that our statement about "some styrene oligomers having binding affinity for hERα" was inaccurate, and that the MCF-7 cell proliferation assay is useless in detecting estrogenicity.

It seems that Ohno et al. have misunderstood our article. We are confident that the results of the MCF-7 cell proliferative assay and the binding assay of styrene oligomers to hERα in our paper are accurate.

The inhibition of fluorescence-labeled $E_2$ binding to hERα by styrene oligomers tested is shown in Figure 3 of our paper (*1*). The inhibition by styrene trimers 1a-phenyl-4e-(1′-phenylethyl)tetralin (ST-3) and 1e-phenyl-4a-(1′-phenylethyl)tetralin (ST-4) was detected at ≥ $5 \times 10^{-7}$ M, and the inhibition by styrene trimers 2,4,6-triphenyl-1-hexene (ST-1), 1a-phenyl-4a-(1′-phenylethyl)tetralin (ST-2), and 1e-phenyl-4e-(1′-phenylethyl)tetralin (ST-5) was detected at ≥ $5 \times 10^{-6}$ M, both sufficiently soluble concentrations. This means that ST-1, ST-2, ST-3, ST-4, and ST-5 bound to hERα at "not high" concentrations. The maximum inhibition by styrene trimers (ST-1, ST-2, ST-3, ST-4, and ST-5) was detected at $5 \times 10^{-5}$ M; this concentration is relatively low. Although the maximum inhibition by styrene dimers 1,3-diphenyl propane, (SD-1), 2,4-diphenyl-1-butene (SD-2), *cis*-1,2-diphenyl cyclobutane (SD-3), and *trans*-1,2-diphenyl cyclobutane (SD-4) was detected at $5 \times 10^{-4}$ M, these styrene trimers and dimers were almost soluble at $5 \times 10^{-5}$ M and $5 \times 10^{-4}$ M, respectively. It is important that the inhibition hardly increased at each 10-times-higher concentration at which chemicals tested were partially insoluble. This result indicates that the soluble chemicals reacted with hER in saturated solution, and insoluble compounds did not influence the binding. In Table 1 of their letter, Ohno et al. did not clarify the solubility of the compounds. It appears that the compounds were dissolved in water because of the extremely low solubility. In our study we dissolved the compounds in DMSO—the styrene dimers at 100,000 µmol/L and the styrene trimers at 10,0000 µmol/L, except for ST-2 (1,000 µmol/L). Ohno et al. should have included the concentrations of the saturating chemicals in the reaction solutions of each method in their Table 1, because when various concentrations of the chemical solvents (DMSO) are added to the reaction solutions, the solubility will become much higher.

If our binding assay indicated false positives in the range of concentrations in which test chemicals were insoluble, the inhibition by 1e,3e,5a-triphenylcyclohexane (ST-6) and 1e,3e,5e-triphenylcyclohexane (ST-7) would also increase, but no binding activity was observed for ST-6 and ST-7 at any concentration tested. This method (Ohno et al.'s Method B) showed an increase in the inhibition of binding by some soluble styrene oligomers but no effect by the same chemicals at insoluble concentrations. Ohno et al.'s Method B showed no $ED_{30}$ values of the styrene oligomers at > 10 µmol/L. Therefore, Ohno et al.'s Method B also indicated no effect by the styrene oligomers at insoluble concentrations except testosterone, 5α-dihydrotestosterone, vitamin $D_3$, and naphthalene. It seems that testosterone, 5α-dihydrotestosterone, vitamin $D_3$, and naphthalene used by Ohno et al. had special characteristics for the competitive binding assay kit (Wako, Osaka, Japan).

The MCF-7 cell proliferation assay is a recognized method for estrogenic screening. Ohno et al. overemphasize other growth factors. All of the styrene oligomers we tested did not have proliferative activity (*1*). ST-6 and ST-7 had no proliferative activity at all, but the proliferative potency of ST-3 and ST-4 was comparable with that of bisphenol A. Moreover we confirmed that OH-tamoxifen, an antagonist, inhibited cell proliferation by ST-1, ST-3, ST-4, ST-5, SD-3, and SD-4 (*2*).

Recently, we reported that ST-1 and ST-4 were estrogenic in the reporter gene assay using MVLN cells established by stable transfection with the luciferase gene (*3*). Moreover we found that some other styrene oligomers were also estrogenic in this reporter gene assay (*2*).

We are confident that our paper (*1*) does not include any inaccurate results.

**Ken-ichi Ohyama**
**Fumiko Nagai**
Tokyo Metropolitan Research Laboratory of Public Health
Tokyo, Japan
E-mail: ohyama@tokyo-eiken.go.jp

### References and Notes

1. Ohyama K, Nagai F, Tsuchiya Y. Certain styrene oligomers have proliferative activity on MCF-7 human breast tumor cells and binding affinity for human estrogen receptor α. Environ Health Perspect 109:699–703 (2001).
2. Ohyama K, Nagai F, Satoh K, Aoki N. Unpublished data.
3. Ohyama K, Nagai F, Satoh K, Uehara A, Ohba M, Uehara S, Aoki N. Hormonal activity of styrene oligomers determined by reporter gene assay using MVLN cells for estrogen and competitive receptor binding assay for androgen. Environ Sci 9:200 (2002).

**Figure 3.** The inhibition of fluorescence-labeled $E_2$ binding to hERα by various concentrations of styrene oligomers. Percent of inhibition was calculated as [1 – (optical density in the presence of competitor) ÷ (optical density in the absence of competitor)] × 100. Each point is the mean ± SD of two independent assays performed in duplicate.
*Significantly different from hormone-free control ($p < 0.01$).

### Corrections and Clarifications

In "3-Chloro-4-(dichloromethyl)-5-hydroxy-2(5H)-furanone (MX) and Mutagenic Activity in Massachusetts Drinking Water" by Wright et al. [*Environ Health Perspect* 110:157–164 (2002)], there are two errors in "Materials and Methods." In lines 12–16 of the second paragraph describing analytical protocol, "700°C" should be "70°C" and "600 mg/L aqueous $NaHCO_3$" should be "600 µg/L 2% aqueous $NaHCO_3$." The correct sentences are as follows:

> The solution was heated to 70°C to accelerate the reaction. The mixture was neutralized by addition of 600 µg/L 2% aqueous $NaHCO_3$ and extracted twice with 600 µL *n*-hexane.

*EHP* regrets the errors.

In "Certain Styrene Oligomers Have Proliferative Activity on MCF-7 Human Breast Tumor Cells and Binding Affinity for Human Estrogen Receptor α" by Ohyama et al. [*Environ Health Perspect* 109:699–703 (2001)], the grids in Figure 3 are incorrect. The corrected figure appears at left. *EHP* regrets any confusion caused by the incorrect grids.

The main point of the letter by Ohno et al. is that styrene oligomers have no estrogenic activity, that our statement about "some styrene oligomers having binding affinity for hERα" was inaccurate, and that the MCF-7 cell proliferation assay is useless in detecting estrogenicity.

It seems that Ohno et al. have misunderstood our article. We are confident that the results of the MCF-7 cell proliferative assay and the binding assay of styrene oligomers to hERα in our paper are accurate.

The inhibition of fluorescence-labeled E_2_ binding to hERα by styrene oligomers tested is shown in Figure 3 of our paper (*1*). The inhibition by styrene trimers 1a-phenyl-4e-(1´-phenylethyl)tetralin (ST-3) and 1e-phenyl-4a-(1´-phenylethyl)tetralin (ST-4) was detected at ≥ 5 x 10^-7^ M, and the inhibition by styrene trimers 2,4,6-triphenyl-1-hexene (ST-1), 1a-phenyl-4a-(1´-phenylethyl)tetralin (ST-2), and 1e-phenyl-4e-(1´-phenylethyl)tetralin (ST-5) was detected at ≥ 5 x 10^-6^ M, both sufficiently soluble concentrations. This means that ST-1, ST-2, ST-3, ST-4, and ST-5 bound to hERα at "not high" concentrations. The maximum inhibition by styrene trimers (ST-1, ST-2, ST-3, ST-4, and ST-5) was detected at 5 x 10^-5^ M; this concentration is relatively low. Although the maximum inhibition by styrene dimers 1,3-diphenyl propane, (SD-1), 2,4-diphenyl-1-butene (SD-2), *cis*-1,2-diphenyl cyclobutane (SD-3), and *trans*-1,2-diphenyl cyclobutane (SD-4) was detected at 5 x 10^-4^ M, these styrene trimers and dimers were almost soluble at 5 x 10^-5^ M and 5 x 10^-4^ M, respectively. It is important that the inhibition hardly increased at each 10-times-higher concentration at which chemicals tested were partially insoluble. This result indicates that the soluble chemicals reacted with hER in saturated solution, and insoluble compounds did not influence the binding. In Table 1 of their letter, Ohno et al. did not clarify the solubility of the compounds. It appears that the compounds were dissolved in water because of the extremely low solubility. In our study we dissolved the compounds in DMSO--the styrene dimers at 100,000 µmol/L and the styrene trimers at 10,0000 µmol/L, except for ST-2 (1,000 µmol/L). Ohno et al. should have included the concentrations of the saturating chemicals in the reaction solutions of each method in their Table 1, because when various concentrations of the chemical solvents (DMSO) are added to the reaction solutions, the solubility will become much higher.

If our binding assay indicated false positives in the range of concentrations in which test chemicals were insoluble, the inhibition by 1e,3e,5a-triphenylcyclohexane (ST-6) and 1e,3e,5e-triphenylcyclohexane (ST-7) would also increase, but no binding activity was observed for ST-6 and ST-7 at any concentration tested. This method (Ohno et al.'s Method B) showed an increase in the inhibition of binding by some soluble styrene oligomers but no effect by the same chemicals at insoluble concentrations. Ohno et al.'s Method B showed no ED_30_ values of the styrene oligomers at > 10 µmol/L. Therefore, Ohno et al.'s Method B also indicated no effect by the styrene oligomers at insoluble concentrations except testosterone, 5α-dihydrotestosterone, vitamin D_3_, and naphthalene. It seems that testosterone, 5α-dihydrotestosterone, vitamin D_3_, and naphthalene used by Ohno et al. had special characteristics for the competitive binding assay kit (Wako, Osaka, Japan).

The MCF-7 cell proliferation assay is a recognized method for estrogenic screening. Ohno et al. overemphasize other growth factors. All of the styrene oligomers we tested did not have proliferative activity (*1*). ST-6 and ST-7 had no proliferative activity at all, but the proliferative potency of ST-3 and ST-4 was comparable with that of bisphenol A. Moreover we confirmed that OH-tamoxifen, an antagonist, inhibited cell proliferation by ST-1, ST-3, ST-4, ST-5, SD-3, and SD-4 (*2*).

Recently, we reported that ST-1 and ST-4 were estrogenic in the reporter gene assay using MVLN cells established by stable transfection with the luciferase gene (*3*). Moreover we found that some other styrene oligomers were also estrogenic in this reporter gene assay (*2*).

We are confident that our paper (*1*) does not include any inaccurate results.

## Corrections and Clarifications

In "3-Chloro-4-(dichloromethyl)-5-hydroxy-2(5H)-furanone (MX) and Mutagenic Activity in Massachusetts Drinking Water" by Wright et al. [*Environ Health Perspect* 110:157-164 (2002)], there are two errors in "Methods." In lines 12-16 of the second paragraph describing analytical protocol, "700°C" should be "70°C" and "600 mg/L aqueous NaHCO_3_" should be "600 µg/L 2% aqueous NaHCO_3_." The correct sentences are as follows:

The solution was heated to 70°C to accelerate the reaction. The mixture was neutralized by addition of 600 µg/L 2% aqueous NaHCO_3_ and extracted twice with 600 µL *n*-hexane.

*EHP* regrets the errors.

**Figure 3:**
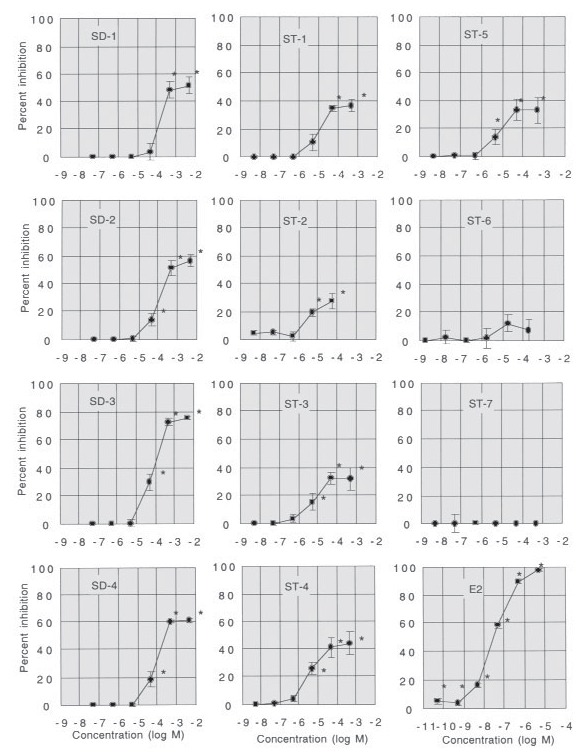
The inhibition of fluorescence-labeled E_2_ binding to hERα by various concentrations of styrene oligomers. Percent of inhibition was calculated as [1 – (optical density in the presence of competitor) ÷ (optical density in the absence of competitor)] × 100. Each point is the mean ± SD of two independent assays performed in duplicate. *Significantly different from hormone-free control (p < 0.01).

In "Certain Styrene Oligomers Have Proliferative Activity on MCF-7 Human Breast Tumor Cells and Binding Affinity for Human Estrogen Receptor α" by Ohyama et al. [*Environ Health Perspect* 109:699-703 (2001)], the grids in Figure 3 are incorrect. The corrected figure appears below. *EHP* regrets any confusion caused by the incorrect grids.
